# The Arab’s Proof

**Published:** 1879-10

**Authors:** 


### THE ARAB'S PROOF.

Some years ago, a Frenchman, who, like many of his countrymen, had won a high rank among men of science, yet who denied the God who is the author of all science, was crossing the great Sahara in company with an Arab guide. He noticed, with a sneer, that at certain times, his guide, whatever obstacles might arise, put them all aside, and kneeling on the burning sands, called on his God.

Day after day passed, and still the Arab never failed, till at last one evening, the philosopher, when he rose from his knees, asked him, with a contemptuous smile:

"How do you know there is a God?"

The guide fixed his eyes on the scoffer for a moment in wonder, and then said solemnly:

"How do I know there is a God?—How do I know that a man, and not a camel, passed my hut last night in the darkness? Was it not by the print of his foot in the sand? Even so," and he pointed to the sun, whose last rays were flashing over the lone desert. "that foot-print is not of a man."

